# Pan-genome diversification and recombination in *Cronobacter sakazakii*, an opportunistic pathogen in neonates, and insights to its xerotolerant lifestyle

**DOI:** 10.1186/s12866-019-1664-7

**Published:** 2019-12-27

**Authors:** Isaiah Paolo A. Lee, Cheryl P. Andam

**Affiliations:** Department of Molecular, Cellular and Biomedical Sciences, University of New Hampshire, Durham, NH 03824 USA

**Keywords:** *Cronobacter sakazakii*, Pan-genome, Core genome, Accessory genome, Recombination, Antibiotic resistance

## Abstract

**Background:**

*Cronobacter sakazakii* is an emerging opportunistic bacterial pathogen known to cause neonatal and pediatric infections, including meningitis, necrotizing enterocolitis, and bacteremia. Multiple disease outbreaks of *C. sakazakii* have been documented in the past few decades, yet little is known of its genomic diversity, adaptation, and evolution. Here, we analyzed the pan-genome characteristics and phylogenetic relationships of 237 genomes of *C. sakazakii* and 48 genomes of related *Cronobacter* species isolated from diverse sources.

**Results:**

The *C. sakazakii* pan-genome contains 17,158 orthologous gene clusters, and approximately 19.5% of these constitute the core genome. Phylogenetic analyses reveal the presence of at least ten deep branching monophyletic lineages indicative of ancestral diversification. We detected enrichment of functions involved in proton transport and rotational mechanism in accessory genes exclusively found in human-derived strains. In environment-exclusive accessory genes, we detected enrichment for those involved in tryptophan biosynthesis and indole metabolism. However, we did not find significantly enriched gene functions for those genes exclusively found in food strains. The most frequently detected virulence genes are those that encode proteins associated with chemotaxis, enterobactin synthesis, ferrienterobactin transporter, type VI secretion system, galactose metabolism, and mannose metabolism. The genes *fos* which encodes resistance against fosfomycin, a broad-spectrum cell wall synthesis inhibitor, and *mdf(A)* which encodes a multidrug efflux transporter were found in nearly all genomes. We found that a total of 2991 genes in the pan-genome have had a history of recombination. Many of the most frequently recombined genes are associated with nutrient acquisition, metabolism and toxin production.

**Conclusions:**

Overall, our results indicate that the presence of a large accessory gene pool, ability to switch between ecological niches, a diverse suite of antibiotic resistance, virulence and niche-specific genes, and frequent recombination partly explain the remarkable adaptability of *C. sakazakii* within and outside the human host. These findings provide critical insights that can help define the development of effective disease surveillance and control strategies for *Cronobacter*-related diseases.

## Background

*Cronobacter sakazakii* (family Enterobacteriaceae; class Gammaproteobacteria) is a motile, gram-negative, rod-shaped opportunistic pathogen that is closely related to more well-known pathogenic genera such as *Enterobacter* and *Citrobacter* [1, 2]. Although *C. sakazakii* has been isolated from various environments, clinical sources, and insects [3–7], many disease cases have been associated with the ingestion of *C. sakazakii*-contaminated dry food products such as powdered milk formula, spices, starches, and herbal teas [8] because of its remarkable ability to tolerate dry conditions [9, 10]. Individuals most susceptible to *C. sakazakii*-induced infections are premature infants and low birth-weight neonates [8, 11], but infections in adults and the elderly have also been reported [12]. *C. sakazakii* infections in neonates and immunocompromised infants are associated with clinical presentations of septicemia, meningitis, and necrotizing enterocolitis [13]. While neonatal infection rates remain low [14, 15], as in the case of the United States where there is one *Cronobacter* infection per 100,000 infants [16], the overall lethality of *Cronobacter* infection can be as high as 27–80% [14, 17], and its impact on the most vulnerable individuals in society makes it a serious health issue. Even when infants survive the infection, different sequelae can potentially threaten their health, including developmental delays, hydrocephaly, and mental retardation [18].

Genomic and evolutionary studies of *C. sakazakii* have been few compared to other bacterial pathogens, but nonetheless reveal important insights that provide a hint to its pathogenic potential and adaptive qualities. Several virulence factors which aid in tissue adhesion, invasion, and host cell injury have been previously reported [19]. An isolate sampled from a female neonate in China was reported to harbor three resistance plasmids IncHI2, IncX3, and IncFIB, which carry multiple resistance genes, including those associated with carbapenems, aminoglycoside, tetracyclines, phenicols, and sulphonamide/trimethoprim [20]. The species exhibits high level of genetic diversity, with some clonal complexes often associated with disease outbreaks. For example, a recent genomic study of 59 contemporary and historical *C. sakazakii* isolates collected from Europe showed remarkable levels of genetic diversity comprising 17 different sequence types (STs) and several isolates harboring genes associated with resistance to multiple classes of antibiotics [21]. Genetic diversity can be high even within an individual patient or a single outbreak event [11]. In the 1994 *C. sakazakii* outbreak in a French neonatal intensive care unit, whole genome phylogeny of 26 isolates revealed four distinct clusters each associated with a distinct ST and the co-circulation of different STs within the same neonate [11]. However, despite its serious health threat to neonates and immunocompromised adults, there has not been a systematic analysis of its population structure, genomic variation and evolutionary history.

In this study, we aim to elucidate the genomic characteristics and phylogenetic relationships of *C. sakazakii* and related species using 285 strains available in the National Center for Biotechnology Information (NCBI). We were particularly interested in determining whether the species is genetically homogenous and if not, to what extent do distinct lineages differ and what processes contribute to this variation? We show that *C. sakazakii* is composed of several deep branching monophyletic lineages that vary in their core allelic and accessory gene content, including many antibiotic resistance and virulence genes. Overall, our results indicate that the presence of a large accessory gene pool, ability to switch between ecological niches, a diverse suite of antibiotic resistance, virulence and niche-specific genes, and frequent recombination partly explain the remarkable adaptability of *C. sakazakii* to survive both within and outside the human host. These findings provide crucial insights on the evolution and pathogenicity of an emerging pathogen that cause fatal neonatal and pediatric diseases, and provide a baseline for the development of effective disease surveillance and control strategies.

## Results

### Characteristics of the *C. sakazakii* pan-genome

**Fig. 1:**
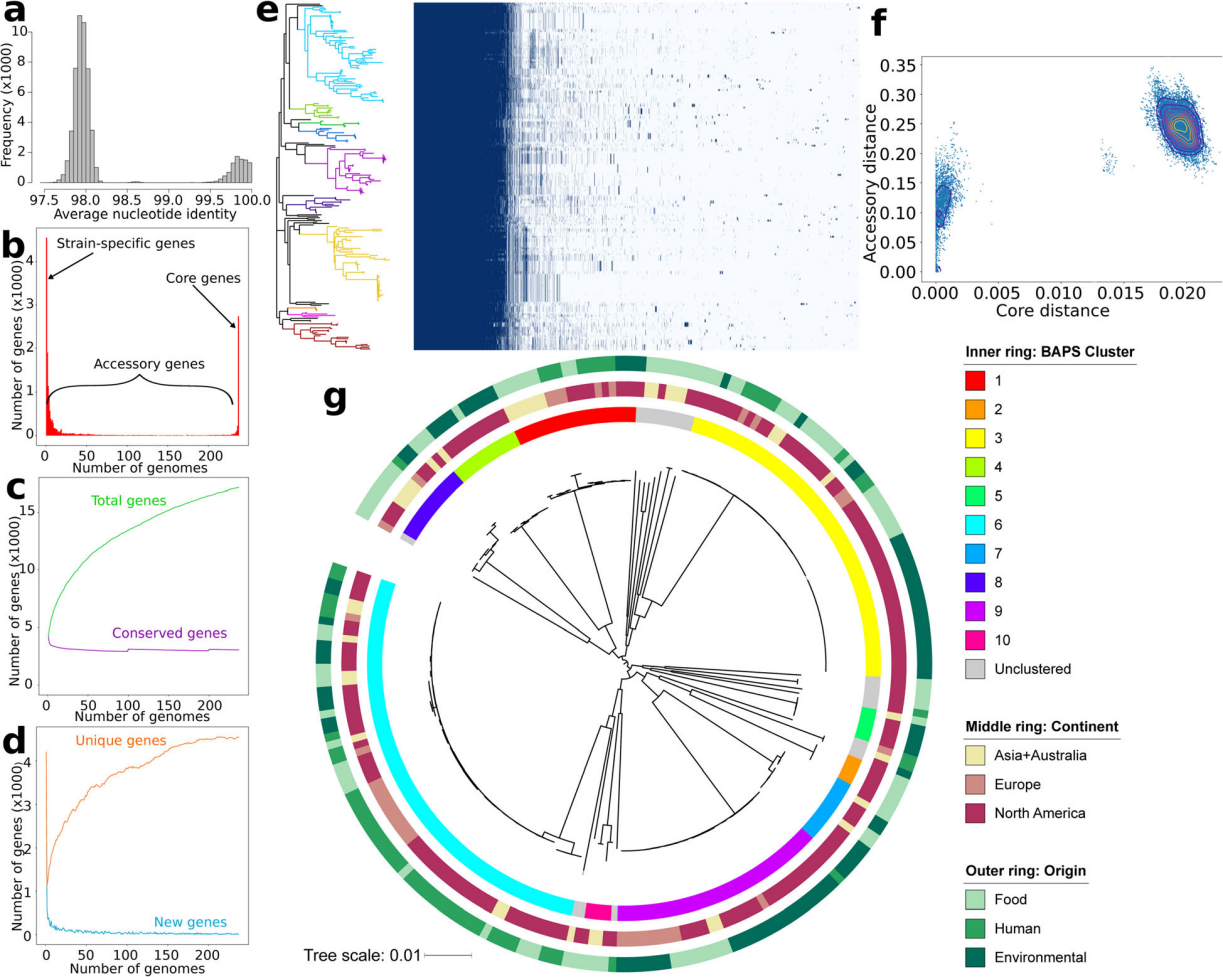
Pan-genome structure and phylogeny of *C. sakazakii*. **a** Distribution of pairwise ANI values. **b** The number of unique genes that are shared by any given number of genomes or unique to a single genome. Numerical values for each gene category are shown in Additional file 6: Table S3. **c** The size of the core genome (purple line) and pan-genome (green line) as more genomes are added. The list of core genes is listed in Additional file 7: Table S4. **d** The number of unique genes, i.e., genes unique to individual strains (orange line) and new genes, i.e., genes not found in the previously compared genomes (light blue line) as more genomes are added. **e** Gene presence-absence matrix showing the distribution of genes present in each genome. Each row corresponds to a branch on the tree. Each column represents an orthologous gene family. Dark blue blocks represent the presence of a gene, while light blue blocks represent the absence of a gene. The phylogeny reflects clustering based on presence or absence of accessory genes. The colors on the tip of each branch reflect the BAPS clustering. **f** Contour plots of pairwise distances between genomes in terms of their core genome divergence (measured by SNP density distance across the core genome) and the difference in their accessory genomes (measured by the Jaccard distance based on the variation in the gene content of their sequences) calculated using popPUNK [24]. **g** The midpoint-rooted maximum likelihood phylogenetic tree was calculated using sequence variation in the core genome alignment. Outer rings show the BAPS cluster, geographical origin, and ecological source. Scale bar represents nucleotide substitutions per site

A total of 313 genomic short read sequences of globally distributed *C. sakazakii* were downloaded from the NCBI Sequence Read Archive (SRA) in October 2018. After checking the quality of genomes using CheckM [22], we further filtered the dataset based on the number of contigs, genome assembly size and number of predicted genes. In all, we used a total of 237 genomes, with the number of contigs ranging from 24 to 443 (median = 68) and assembly size ranging from 4.14–4.8 Mb (Additional file 4: Table S1). Calculation of the genome-wide average nucleotide identity (ANI) for all pairs of genomes indicates that all genomes are within the minimum 95% threshold that defines a species [23] (Fig. 1a; Additional file 5: Table S2).

The number of predicted genes per genome ranges from 3739 to 4535 (mean = 4156). We used Roary [25] to calculate the *C. sakazakii* pan-genome, which refers to the sum of all genes present in a species or any group of genomes under study [26] (Additional file 6: Table S3; Fig. 1b). The number of genes present in at least 99% of the strains, also referred to as core genes, is 3072. The number of soft core genes, which are present in at least 95% but less than 99% of the strains, is 273. The combined core and soft core genes (*n* = 3345 genes; Additional file 7: Table S4) constitute only 19.5% of the entire species’ pan-genome (*n* = 17,158 genes). The size of the core genome that we identified is consistent with that found in 59 *C. sakazakii* isolates from a 2017 multicenter study from 11 countries in Europe which reported 2831 core genes [21]. The core genome makes up 73.81–86.46% (mean = 79.28%) of each *C. sakazakii* genome. The accessory genome is composed of the shell genes which are present in at least 15% but less than < 95% of the strains (*n* = 1458 genes) and cloud genes which are present in less than 15% of strains (*n* = 12,355 genes representing 72.0% of the pan-genome). It is notable that many accessory genes are unique to a single strain (4545 genes, representing 26.49% of the pan-genome). In microbes, large accessory genomes and high numbers of strain-specific genes are often associated with frequent gene gain and loss [27–29].

We next estimated how many new genes are discovered as more and more strains are sequenced [26, 30]. The pan-genome of *C. sakazakii* is open, which means that future sequencing of genomes will likely result in finding previously unidentified genes (Fig. 1c). The existence of a large and open pan-genome is often associated with organisms that are able to inhabit diverse habitats (e.g., present in both soil and eukaryotic host, present in multiple host species) or those that frequently undergo horizontal gene transfer with other taxa [31, 32]. We also found that the core genome declines in size as more genomes are added. Finally, we also show that the number of novel genes and unique genes continue to rise as additional genomes are included (Fig. 1d). The distribution of accessory genes however varies among strains (Fig. 1e). We also calculated the genomic fluidity φ, which estimates the number of identical gene families that are shared between genomes [33]. *C. sakazakii* has a genomic fluidity value of 0.875 (standard deviation, s.d. = 0.309), which indicates that 87.5% of the genes are unique to their host genome and the remaining 12.5% are shared between genomes. Overall, these results show that strains of *C. sakazakii* have access to a large accessory genome pool, with individual strains each having a unique repertoire of potentially useful genes.

To gain insight on how the accessory genome has diverged in relation to the core genome, we used PopPUNK which employs pairwise nucleotide k-mer comparisons to distinguish shared core sequence and gene content [24]. Results show a discontinuous distribution of pairwise genomic distances, with more genetically similar genomes found tightly clustered near the origin of the graph, while larger genetic distances are concentrated away from the origin (Fig. 1f). This discontinuity in the two sets of points is indicative of the presence of multiple genetically distinct clusters that are diverging in both core sequences and accessory gene content. Overall, these data show that *C. sakazakii* is composed of many genetically distinct lineages that can be distinguished in their core and accessory genome divergence patterns.

To investigate the genetic structure of the *C. sakazakii* dataset, we extracted and concatenated the sequences of the 3345 core genes using RhierBAPS [34]. The clustering analysis started with 20 initial populations until it converged to a local optimum, resulting in 11 identified primary sequence clusters (called SCs), of which one consisted of unclustered strains that cannot be classified in any of the SCs. The ten SCs range in size from 4 to 66 genomes per cluster (Fig. 1g). There are several deep branching monophyletic lineages indicative of ancestral diversification. Three large SCs (SCs 3, 6, and 9) constitute majority of the dataset, but we also found numerous highly diverse SCs that are present in low frequency. There is relatively little structure related to geographical or ecological sources. Almost all SCs contain strains from different continents and origins (food, human, or environment), which shows that none of the lineages appear to be specifically associated with any one niche. Frequent switching between ecological niches appears to be common, as observed from the intermingling of strains from different sources within each SC and throughout the phylogenetic tree.

### Within-species variation in the core and accessory genomes of *C. sakazakii*

Considering the phylogenetic and ecological diversity of *C. sakazakii* strains in this dataset, we further examined the mutations that contribute to this variation within the species. We first compared the three largest SCs (SCs 3, 6, and 9) by estimating the number of core single nucleotide polymorphisms (SNPs) within each SC (Additional file 1: Fig. S1). We found significant differences among them (*p* < 0.001, ANOVA), with SC 6 having the highest mean SNP distance (number of pairwise SNPs = 1249.81, s.d. 1538.26) followed by SC 3 (265.63, s.d. 468.54) and SC 9 (216.42, s.d. 89.59). We next examined pairwise distances between strains grouped by source (food, human, environment) (Additional file 1: Figure S1). We also found significant differences among the three (p < 0.001, ANOVA), with food strains having the highest mean SNP distance (51,248.27, s.d. 17,378.93) followed by environmental strains (46,454.3, s.d. 22,034.74) and human strains (32,924.87, s.d. 28,083.43).

We also calculated the ratio of substitution rates at each nucleotide site by estimating the dN/dS ratio of all core genes, thereby providing insights to the strength of selection acting on the core genome of *C. sakazakii* (Additional file 1: Figure S1 and Additional file 8: Table S5). The ratio dN/dS is commonly used metric to detect selection acting on a gene, with dN/dS > 1 indicating positive selection and dN/dS < 1 indicating purifying selection [35]. We found evidence for positive selection in 16 genes, of which nine have hypothetical functions. Five genes have dN/dS approaching infinity, indicating either positive, diversifying selection on amino acids or strong purifying selection on synonymous codons [35]. The gene *macA*, which encodes a macrolide-specific efflux protein [36] and has been reported in the type strain *C. sakazakii* ATCC BAA-894 [37], has a dN/dS = 3.95. Other genes with dN/dS > 1 include *yaiY* (1.96; inner membrane protein), *elfA* (1.84; fimbrial subunit), *atpC* (1.83; ATP synthase), *kdul* (1.70; hexuronate metabolism) and *livK* (1.51; leucine-specific-binding protein), although these functions are based on *Escherichia coli* and their specific functions in *C. sakazakii* remain unclear. Two genes are notable however. The gene *elfA* codes for a fimbrial subunit protein, and fimbriae-related proteins are known to be virulence factors in *Cronobacter* and other Enterobacteriaceae, promoting attachment and aggregation on biotic and abiotic surfaces [38, 39]. The gene *kdul* is a component of the hexuronate metabolism pathway in *E. coli* which converts the carbohydrates galacturonate and glucuronate under osmotic stress conditions in mice fed with a lactose-rich diet, playing an essential role in bacterial adaptation to lactose-mediated osmotic stress [40]. The gene with the highest dN and dS values is *icsA*, which encodes an outer membrane autotransporter protein known to be a key virulence factor in *Shigella flexneri* and functions to mediate intracellular motility, intercellular spread and adhesion [41].

We next sought to identify the accessory genes that are unique to each SC or ecological source (food, human, or environment). Using the pan-genome output of Roary, we first searched for genes that are exclusive to a specific SC or ecological source. The number of accessory genes that are SC-exclusive range from 64 in SC2 to 1,871 in SC6 (Additional file 2: Figure S2, Additional file 9: Table S6), while source-exclusive accessory genes total to 3,297, 2,570 and 1,968 in human, food and environmental sources, respectively (Additional file 2: Figure S2; Additional file 10: Table S7). Using PANTHER [42], we next examined the functional classification of both the genes present in each SC and the genes exclusive to each SC, using the full set of genes in the pan-genome as a reference (Additional file 11: Table S8). We obtained significant results only for three SCs. The genes exclusive to SC 1 were enriched for genes involved tryptophan biosynthesis, indole biosynthesis, and amine metabolism. The genes exclusive to SC 4 were enriched for genes associated with nucleoside-triphosphatase, pyrophosphatase and hydrolase activities. The genes exclusive to SC 5 were enriched for biofilm formation. These differences between SCs suggest fine-scale variation in adaptive potential among some lineages and may explain the findings from previous studies that report that certain *C. sakazakii* lineages are often associated with disease outbreaks [11, 43]. However, it is curious that none of the three major SCs displayed significant functional enrichment. We also classified the functions of genes exclusive to each ecological source. We detected enrichment of genes involved in proton transport and rotational mechanism in human-exclusive accessory genes. In environment-exclusive accessory genes, we detected enrichment for those involved in tryptophan biosynthesis and indole metabolism. However, we did not find significantly enriched gene functions for those genes exclusively found in food strains. We also did not detect significant depletion of genes associated with the SCs or source. These source-associated differences may therefore partly explain the ability of *C. sakazakii* to adapt to different ecological niches both outside and inside the human host, and the repertoire of niche-associated genes will be instrumental in their adaptive capability. We predict that certain lineages and strains are more able to adapt and are frequently found in either human or environmental settings, although experimental evidence and more extensive sampling is needed to verify this.

### Antibiotic resistance and virulence in *Cronobacter*

While rare, non-*sakazakii* species have been reported to potentially cause morbidity and life-threatening complications in infants and adults [12, 44] and we therefore included them in our analyses (*n* = 48 genomes; Additional file 4: Table S1). Initially considered a unique group within the genus *Enterobacter*, *Cronobacter* species have had a convoluted history of misclassification and multiple instances of re-naming [45]. To date, there are seven recognized species of *Cronobacter*, with *C. sakazakii* being the most clinically significant. However, correct species identification of *Cronobacter* species remains a challenge. In this study, six species of *Cronobacter* were included (*Cronobacter dublinensis, Cronobacter malonaticus, Cronobacter muytjensii, Cronobacter turicensis* and *Cronobacter universalis*). *Cronobacter condimenti* was not included because of lack of sequenced genomes in the NCBI database.

**Fig. 2:**
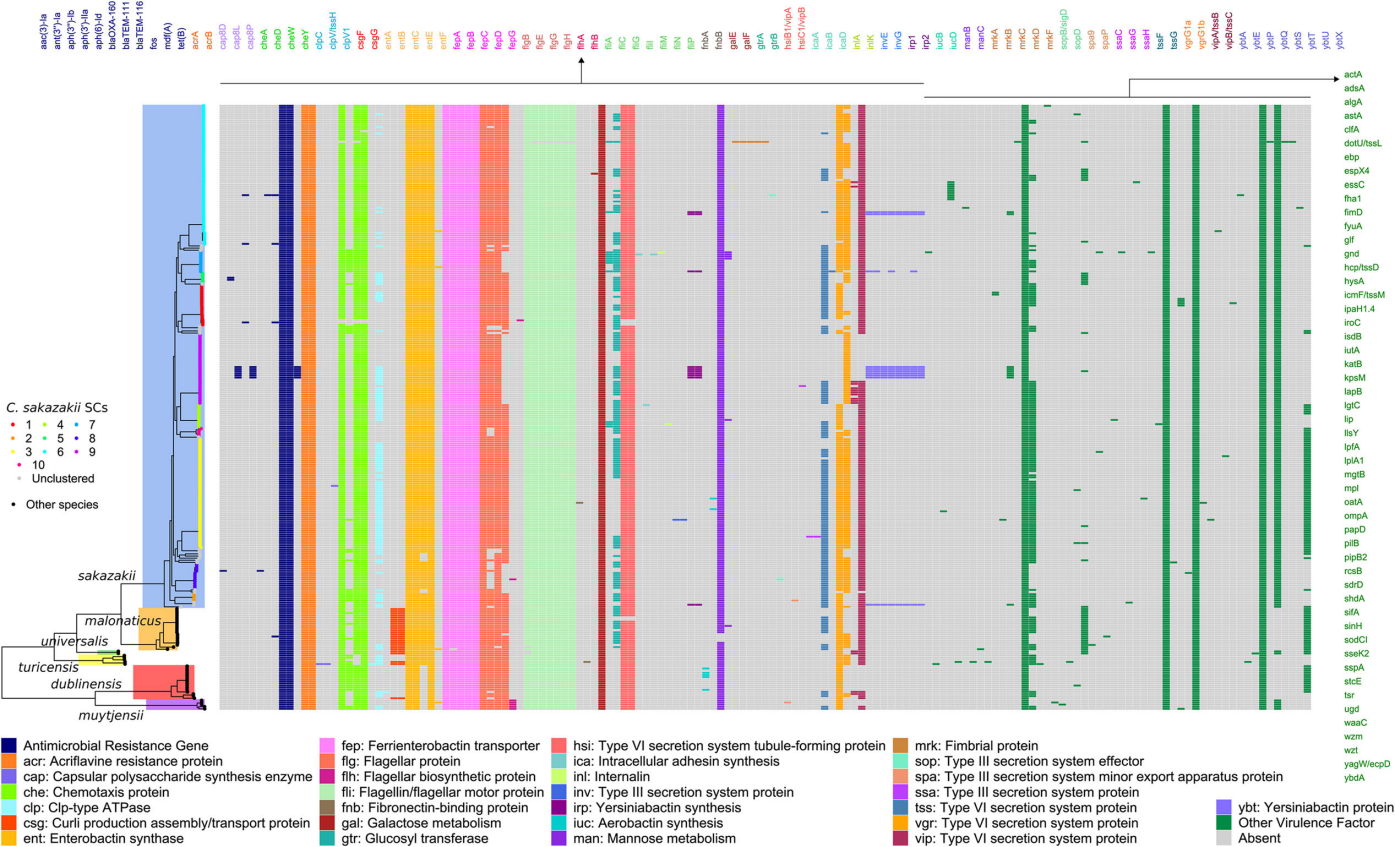
Distribution of antibiotic resistance and virulence genes in *C. sakazakii* and related species. Columns and gene names are colored according to related functions, except for those with distinct functions (colored in green). The midpoint-rooted maximum likelihood phylogenetic tree was calculated using sequence variation in the core genome alignment of the entire genus (*n* = 1942 genes). Scale bar represents nucleotide substitutions per site

Studies of recent infections and disease outbreaks indicate that *C. sakazakii* and related species exhibit resistance to certain antibiotics [20, 46]. We sought to systematically examine the presence and distribution of horizontally acquired genes that confer antibiotic resistance and encode virulence factors across the entire *Cronobacter* dataset. Using the program ABRicate, we found that the most common horizontally acquired antibiotic resistance genes (in contrast to resistance due to chromosomal mutations) across the genus were *fos* and *mdf(A)*, which were detected in all genomes (Fig. 2; Additional file 12: Table S9). The *fos* gene encodes resistance against fosfomycin, a broad-spectrum cell wall synthesis inhibitor [47]. It has been previously reported in *Cronobacter* [46] and is also known to be widespread in many genera of gram-negative bacteria [48]. The gene *mdf(A)* has been well characterized in *E. coli* and is known to encode a multidrug efflux transporter with an unusually broad pattern of drug specificities [49]. However, it remains unclear if this transporter confers resistance to the same spectrum of antibiotics in *Cronobacter*. Other antibiotic resistance genes detected but at lower frequencies are those confer resistance against aminoglycosides, beta-lactams, and tetracyclines. We also detected genes *acrA* and *acrB* in all genomes. In *E. coli*, the AcrB-AcrA fusion protein acts as a multidrug efflux transporter [50]. The genes *fos, acrA* and *acrB* have been previously detected in C. *sakazakii* strains SP291 and type strain ATCC BAA-894 isolated from powdered infant formula [37]. Future work should therefore focus on understanding the origins of these acquired resistance genes and developing effective detection methods of multidrug resistant phenotypes.

We also used ABRicate to identify the variety of virulence genes in *Cronobacter* genomes (Fig. 2; Additional file 12: Table S9). The most frequently detected genes are those that encode proteins associated with chemotaxis, enterobactin synthesis, ferrienterobactin transporter, type VI secretion system (T6SS), galactose metabolism, and mannose metabolism. Other virulence genes detected in all or majority of genomes are *gnd* (6-phosphogluconate dehydrogenase), *ompA* (outer membrane protein A essential for adhesion to and invasion of the cell), *rcsB* (transcriptional regulatory protein), *tsr* (methyl-accepting chemotaxis protein), and *waaC* (heptosyltransferase involved in the synthesis of lipolysaccharides). It is not surprising that these genes are prevalent throughout *C. sakazakii* and related species. *Cronobacter* can enter human intestinal cells and in rare cases invade the blood brain barrier [38]. Chemotaxis, flagellar proteins and outer membrane proteins are therefore critical in the attachment to and invasion of the intestinal cells [51, 52]. The ability to acquire and metabolize nutrients is also crucial to surviving outside of the human host, enabling the bacterium to utilize limiting nutrients such as iron from powdered milk formula and dried food products. Metabolism of the sugars galactose and mannose are also critical to surviving in these environments so they can take advantage of these nutrients. Lastly, T6SS-associated proteins are widely distributed in gram-negative bacteria and this secretion system is used as a molecular weapon against hosts, predators and competitors [53]. In *Cronobacter*, T6SS likely plays a role in cellular invasion, adherence, cytotoxicity, and growth inside macrophages [38].

Overall, we show that a multitude of genes that encode resistance and virulence factors are widespread not just in *C. sakazakii* but also in other *Cronobacter* species. Many if not all genomes carry genes that allow them to grow and survive inside the human host as well as in dry food products outside of their host. However, we did not find evidence for resistance or virulence genes that are associated with specific lineages or species.

### Recombination in *C. sakazakii* genomes

Bacteria can receive DNA fragments from other species and integrate them into their chromosomes through recombination [54, 55]. The process of recombination plays a fundamental role in the evolution of many bacterial pathogens and has been implicated in the emergence of highly virulent and drug resistant lineages [54, 55]. Here, we sought to determine the extent of recombination in *C. sakazakii* because this process may likely contribute to its genomic variation and evolutionary history. Here, we focus only on homologous recombination of both core genes and shared accessory genes, and not on other mechanisms of recombination (e.g., illegitimate, site-specific). Recombination that brings in novel DNA sequences, as in the case of strain-specific genes and acquired antibiotic resistance genes described above, are likely mediated by mobile genetic elements and are not included in the analyses below.

**Fig. 3:**
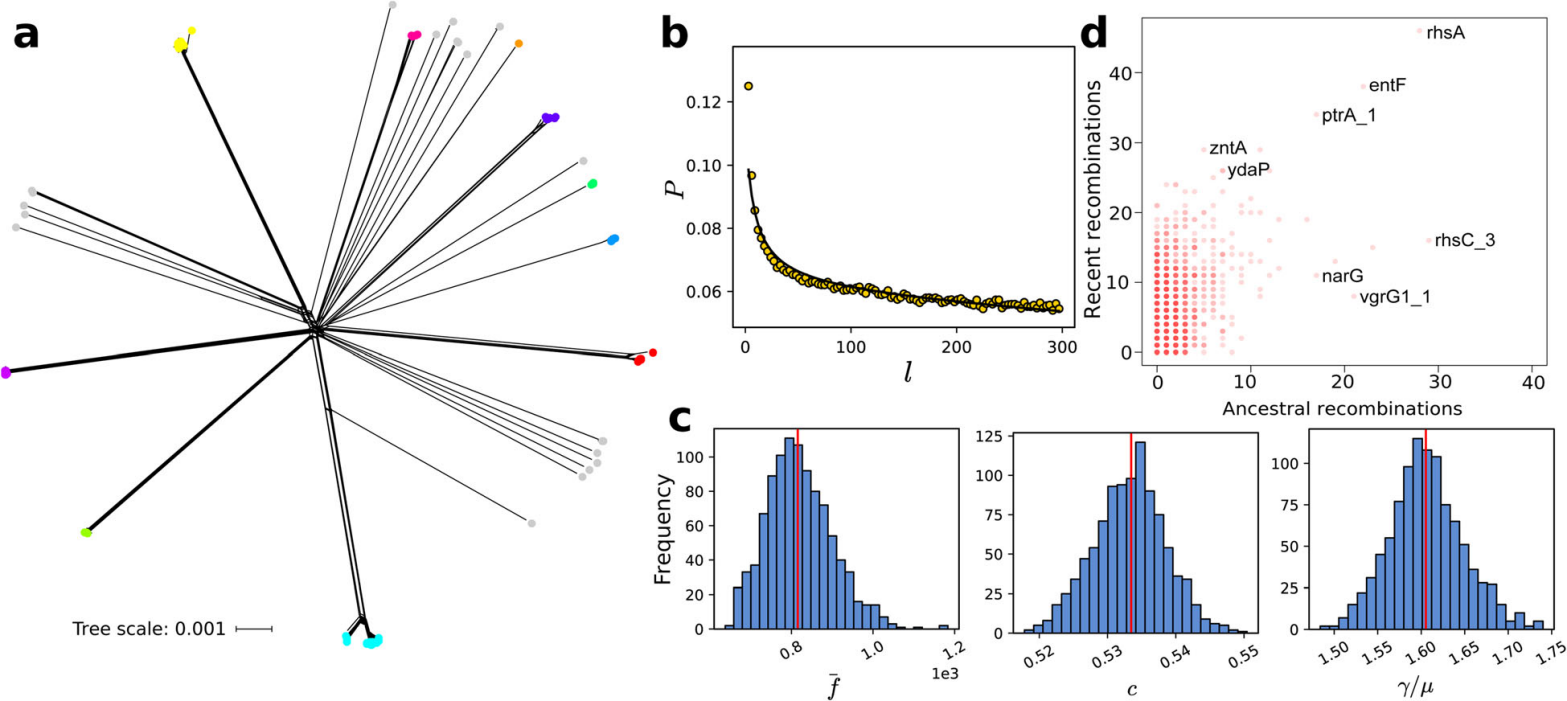
Recombination in *C. sakazakii*. (**a**) A phylogenetic network of the core genome generated using SplitsTree. Scale bar represents nucleotide substitutions per site. Colored dots represent BAPS clusters and are identical to those in Fig. 1a. (**b**) Correlation profile (circles) calculated from the core genomic alignment by mcorr. Model fit is shown as a solid line. (**c**) Frequency histograms showing the distributions of the three recombination parameters for all pairs of genomes. The red vertical lines indicate the means. (**d**) Genes that have undergone recent or ancestral recombination. Horizontal axis shows the estimated number of ancestral recombinations, and vertical axis shows the estimated number of recent recombinations. For visual clarity, names of some of the genes with known function are shown

Under the null hypothesis of no recombination, we calculated the pairwise homoplasy index (PHI) statistic [56] and detected evidence for significant recombination in the core genome (*p*-value = 0.0). Recombination in *C. sakazakii* core genome can be visualized using NeighborNet implemented in SplitsTree4 [57], which incorporates reticulations due to non-vertical inheritance in phylogenies (Fig. 3a). This observation is further supported by results from calculating the probability that a pair of genomes differs at one locus conditional on having differences at the other locus using the program mcorr [58]. The correlation profile for *C. sakazakii* exhibits a monotonic decay (Fig. 3b), which shows that recombination causes pairs of sequences to become identical over random DNA blocks [58]. Overall, the results of the Splitstree, PHI test and correlation profile analyses all provide evidence that recombination has had an impact on the evolutionary history and core genome structure of *C. sakazakii*.

We next sought to precisely quantify the impact of recombination on the genetic diversity of *C. sakazakii*. To achieve this, we again used the program mcorr to calculate different recombination parameters (Fig. 3c; Additional file 13: Table S10). The mean fragment size (f̅) of a recombination event was estimated to be 815.559 bp (s.d. = 80.203). The recombination coverage (c) indicates the fraction of the genome whose diversity was derived from recombination events since its last common ancestor and ranges from 0 (clonal evolution) to 1 (complete recombination) [58]. We estimate this parameter to be 0.53346 (s.d. = 0.00529), which means that 53.3% of the genome has had a history of recombination. Lastly, the ratio γ/μ, which gives the relative rate of recombination to mutation, was estimated to be 1.6054 (s.d. = 0.04224). These values are comparable to *Acinetobacter baumannii*, another well-known pathogen that is closely to *Cronobacter* and is a member of Gammaproteobacteria, which has a f̅, c and γ/μ of 860 bp, 0.40 and 1.3, respectively [58].

We hypothesize that certain genes are more often recombined than others, which may reflect their ecological importance. To identify the specific genes that are frequently recombining, we ran fastGEAR [59] on individual sequence alignments of core and shared accessory genes. We found that, of the 17,158 genes that comprise the pan-genome, a total of 2991 genes have had a history of recombination (Fig. 3d; Additional file 14: Table S11). A total of 2843 genes were involved in recent recombination and 1097 genes in ancestral recombination. Many of the most frequently recombined genes identified by fastGEAR are associated with metabolic growth, survival, and toxicity. Among the metabolism genes, the *ydaP* genes is a homologue of *E. coli* pyruvate oxidase and has been suggested to convert pyruvate to acetyl-P [60] thereby contributing to aerobic growth efficiency [61]. The *narG* gene encodes for the nitrate reductase 1 alpha subunit, which functions in nitrogen metabolism [37]. It has been found in *Cronobacter*, but its presence has not been shown to change the ability of a strain to metabolize nitrogen [37]. The gene *entF* encodes for the serine activating enzyme in enterobactin biosynthesis and is part of a gene cluster that functions in iron acquisition [62]. Enterobactin is a high affinity siderophore (iron chelator) that is produced and secreted specifically in response to iron deficiency [63]. The *zntA* gene codes for a zinc/cadmium/lead-transporting P-type ATPase, which has been found to confer resistance to zinc, cadmium, and lead in *E. coli* [64]. This stress response gene has been found in *C. sakazakii* resistance plasmids [20]. The *ptrA* gene codes for the metalloendopeptidase pitrylysin, which is involved in insulin degradation in *E. coli* The genes *rhsA* and *rhsC* are part of the complex 5-member *rhs* family (which stands for rearrangement hot spots) and was previously identified as having a core open reading frame that provided homology for a frequent but unequal intrachromosomal recombinational event [65, 66]. In *Enterobacter cloacae, rhsA* causes growth inhibition of other bacteria via T6SS [67]. In *Vibrio cholerae*, *vgrG1* encodes thee valine-glycine repeat protein G and has been shown to function as a toxin through actin cross-linking [68]. In *E. cloacae* and *Pseudomonas aeruginosa*, VgrG has been shown to function in the delivery of T6SS effectors [67]. Furthermore, it has recently been shown that T6SS-2 is a virulence factor in *C. sakazakii* [69]. Other *Cronobacter* species also show evidence of recombination and likely contributes to their pathogenicity (Additional file 3: Figure S3; Additional file 15: Table S12). Frequent recombination is often reported to accelerate adaptation in bacterial populations, enabling survival in rapidly changing environments [70]. Hence, for *C. sakazakii*, frequent recombination of these genes likely confers a benefit to a lifestyle that requires rapid adaptation and metabolic growth to disparate ecological niches (human, food, environment).

## Discussion

Although rates of *C. sakazakii* infection in neonates remain low, its impact on the most vulnerable individuals in society makes it a serious health issue. Several *C. sakazakii* outbreaks in infant and adult patients have been reported in previous years [8, 16, 21]. However, large-scale genomic studies, which can provide crucial information on a pathogen’s genetic diversity, ecological adaptation, antibiotic resistance and virulence have been noticeably lacking in *C. sakazakii*. While previous genomic studies have revealed important insights into its ecology and evolution, only a few genomes are typically compared. Hence, an important step forward in understanding the mechanisms that shape microbial genome dynamics is to examine populations that represent clusters of close relatives within and between environments. The emerging field of population genomics offers unprecedented sensitivity for the detection of rare genotypes, vastly improved resolution for evolutionary studies, and direct sequencing of functionally and ecologically relevant loci [71–73]. The open pan-genome of *C. sakazakii* implies that more and more novel genes will be discovered with the addition of more sequenced genomes and can therefore be used as a springboard for developing future experimental and functional assays. We present a systematic, population-level analysis of 285 genomes from a variety of sources to gain insight into the ecological differentiation and associations of *C. sakazakii*. This study also offers a deeper understanding of the evolutionary mechanisms that create and maintain diversity within and between *C. sakazakii* populations.

There are two main findings in this study. First, *C. sakazakii* is composed of multiple distinct lineages that greatly vary in their core and accessory genomic characteristics. Results indicate that within-species genomic diversity is due to the presence of multiple deep branching lineages indicative of ancestral diversification. The global *C. sakazakii* population is dominated by three major lineages (SCs 3, 6, and 9) and within each SC, genomes display very similar core genome sequences indicative of recent but rapid diversification. We detected core genes that have undergone either positive or diversifying selection, which include those associated with virulence (e.g., motility) and ecological adaptation (e.g., osmotic stress). There are also numerous highly diverse SCs that are present in low frequency, which further expands the genetic diversity of the species. The existence of multiple co-circulating but genetically diverse lineages have been reported in other pathogenic bacteria [74], and may contribute to the adaptability of the entire species. The intermingling of genomes from different ecological sources, whether it was food, human, or environmental, suggests that there is not one specific lineage that is often associated with each source and that they can easily switch between different environments. We also did not find any evidence that certain acquired antibiotic resistance and virulence genes are associated with specific phylogenetic groups or sources. However, we did find significant functional differences in the accessory genes that are exclusively found in some SCs or ecological source. We interpret these results from the phylogenetic distribution and functional classification to mean that any one lineage can potentially inhabit multiple environments, but some are more likely to succeed in specific environments because of the niche-specific genes they carry. This is particularly notable in our results that show enrichment of genes involved in chemotaxis and flagella in human-associated strains, while nutrient synthesis and metabolism are enriched in environmental strains. However, we did not find significant functional enrichment in the three major SCs, which may imply that they likely have similar adaptive potential. One important line of inquiry for future research is therefore determining the genetic and ecological factors that drive the dominance of certain lineages (SCs 3, 6, 9) in the population and whether these same lineages are also the major cause of infections, or that their prevalence is simply due to sampling bias. Future work also requires a more extensive and consistent sampling from a multitude of sources (e.g., countries, age groups of patients, clinical presentations, food products, animals) to precisely define how the *C. sakazakii* gene pool is distributed across the spatiotemporal landscape. Elucidating the frequency, mechanisms and drivers of niche switching in this pathogen is also critical to making accurate predictions of the impact of foodborne *Cronobacter*-related infections and disease outbreaks. Population genomics, which involves analyses of hundreds or thousands of genomic sequences from microbes that inhabit different hosts or environments, will be instrumental in advancing our knowledge about the adaptive potential of this pathogen as has been done in other well-studied bacterial pathogens (e.g. *Streptococcus pneumoniae, Staphylococcus aureus*) [75]. This study provides a first step to developing a population-level framework to precisely define the range of *C. sakazakii*’s adaptive strategies in difficult conditions and the boundaries of its ecological niches.

Second, in addition to ancestral diversification and group-specific functional differences, recombination has greatly contributed to shaping the population structure of *C. sakazakii*. While its recombination rate is comparable to other pathogenic Gammaproteobacteria [58], it is notable that the most frequently recombining genes are those associated with metabolic growth, survival, and toxicity, all of which can aid in survival within the human host and in extreme environments and which can be disseminated rapidly to other members of the population. The large number of strain-specific genes and horizontally acquired antibiotic resistance genes further supports frequent gene gain and loss, likely through mobile genetic elements. Pathogens that can thrive in ecologically diverse settings have in place a plethora of systems, including frequent recombination, to respond to changes in their surroundings. Recent studies of large-scale sequencing of bacterial genomes indicate that rates of recombination can vary dramatically within a species [76, 77]. In these studies, certain lineages have been reported to act as hubs of gene flow, whereby they are more often involved in DNA donation and receipt compared to other closely related lineages [76]. These differences are often not trivial because such fine-scale variation may define major functional, clinical, ecological and adaptive potential. For example, hyper-recombinants have been reported to exhibit significantly higher levels of antibiotic resistance [70, 78]. Recombination hubs in bacterial populations may also allow certain rare genes to be maintained in the population and not be lost, thereby allowing the population or species as a whole to benefit from these rare genes when needed. Future work in *C. sakazakii* should therefore examine whether certain lineages exhibit higher than average rates of recombination, whether through investigations of naturally occurring isolates or experimental evolution approaches, and the barriers that reduce recombination between certain populations (e.g., lack of niche overlap, geographical distance, or intrinsic genetic mechanisms such as restriction-modification enzymes [79]). Moreover, it is also imperative that a deeper investigation of the different mechanisms of recombination (e.g., homologous, illegitimate, site-specific, mediated by mobile genetic elements, replacement versus additive) is needed, focusing on their relative contributions in shaping the genome structure and evolution of *C. sakazakii*.

Limitations of the present work stem mainly from the biases in sampling schemes and genome sequencing studies of *Cronobacter*. Information on the diversity, pathogenicity, and virulence of other *Cronobacter* species obtained from various sources is still relatively scarce and fragmentary, although they have been reported to be also implicated in serious infections [3, 46]. Hence, genomic comparison of different species proves to be challenging. To date, evaluating inter-species differences in *Cronobacter* relies mainly on representative or type strains. Another limitation is that detection of antibiotic resistance, virulence and other ecologically relevant genes depends mainly on the composition of current databases that are used for comparing sequence similarities. It is probable that *C. sakazakii* harbors novel mechanisms of resistance and virulence or has novel cellular targets that may be absent in other well studied bacterial pathogens. Its large repertoire of strain-specific genes may hold valuable insights into these new functions. We expect that our findings will provide critical information to mine these genomes for novel functions and traits. Niche-adaptive genes involved in chemotaxis, enterobactin synthesis, ferrienterobactin transporter, T6SS, galactose metabolism, and mannose metabolism as well as positively selected core genes will be an excellent starting point in functional assays in the future. Lastly, we underscore the need to undertake population genomics approaches to elucidate the genetic diversity of *C. sakazakii* and ensure the development of accurate detection methods, effective disease control and reliable microbial source tracking of contaminated foods.

## Conclusions

In summary, we show that *C. sakazakii* is phylogenetically and genomically diverse. There are at least ten deep branching monophyletic lineages indicative of ancestral diversification, each of which appears to have rapidly diversified in recent times. The presence of a large accessory gene pool, ability to switch between ecological niches, a diverse suite of antibiotic resistance, virulence and niche-specific genes, and frequent recombination partly explain the remarkable ecological versatility and xerotolerant lifestyle of *C. sakazakii*. Results from this study are expected to inform molecular diagnostic tools that can be used in implementing successful surveillance programs and in the control and prevention of *Cronobacter*-related foodborne illnesses.

## Methods

### Dataset

A total of 313 *Cronobacter* genomes available in October 2018 were downloaded from the NCBI SRA database. Accession numbers and information (total read length, annotation statistics, and metadata) are shown in Additional file 4: Table S1. The sequences were trimmed using Trimmomatic v.0.36 [80] with a four-base sliding window, a minimum PHRED score of 15 and a minimum length of 35. The sequences were assembled using SPAdes v3.10.0 [81] with default parameters. Two misassembled genomes (SRR7235683 and SRR7439201) were removed from analysis. We assessed the quality of the genomes using CheckM v.1.0.13 [22] to exclude genomes with less than 90% completeness (SRR7419954) and greater than 5% contamination (SRR7367482, SRR7419954, DRR015813, DRR015986, DRR015987, SRR944696, DRR015812). Finally, we removed those assemblies with > 500 contigs (SRR7235892, SRR7419951, SRR7419962, SRR7439218, DRR015912). The genomes were annotated using Prokka v.1.12 with default parameters [82]. We carried out genome re-assembly and re-annotation to maintain consistency in gene assignments.

To determine the degree of genomic relatedness, we calculated pairwise ANI values using the program FastANI v.1.1 [23] and were visualized using an heatmap generated by the R package gplots (https://cran.r-project.org/web/packages/gplots/index.html). A highly divergent cluster with only 81% identity compared to the other genomes was removed from downstream analysis. This cluster included genomes corresponding to SRA run numbers ERR474280, ERR474434, ERR474430, ERR474435, ERR474449, ERR474436, ERR474450, ERR474458, ERR486105, ERR474461, ERR486111, ERR486181, ERR502554, and ERR486184. While these were originally classified as *C. sakazakii* in NCBI, the low ANI values suggest that they are likely members of another genus. This is not unexpected given the history of misclassification of *Cronobacter* with the closely related *Enterobacter* [45]. We further confirmed this by comparing the sequences encoding the 16S rRNA gene of the 14 genomes with sequences in the non-redundant database of NCBI using BLAST [83]. All 14 sequences are most closely similar to *Enterobacter hormaechei* and *E. cloacae*. Strains that were highly similar to those of another named species but not to strains labelled with their original species were reclassified for downstream analysis. These included DRR015985 assigned from *C. dublinensis* to *C. sakazakii*, DRR015912 assigned from *C. malonaticus* to *C. sakazakii*, DRR015811 assigned from *C. dublinensis* to *C. malonaticus*, and SRR7367486 assigned from *C. malonaticus* to *C. turicensis*. The final dataset included *C. sakazakii* (*n* = 237), *C. malonaticus* (*n* = 20), *C. dublinensis* (*n* = 16), *C. turicensis* (*n* = 5), *C. muytjensii* (n = 5), and *C. universalis* (n = 2). Overall, we used a total of 285 genomes in this study.

### Pan-genome and phylogenetic analyses of *Cronobacter*

Pan-genome and phylogenetic analyses were done as previously described [84]. To summarize, core and accessory genes were identified using Roary v.3.12.0 with default settings [25] and sequences of individual gene families were aligned using MAFFT [85]. We used the program micropan [86] implemented in R [87] to calculate the pan-genome’s genomic fluidity (φ) which measures genome dissimilarity as a function of the degree of overlap in gene content [33]. The gene sequence alignments of each core gene family were concatenated to give a single core alignment, which was used to generate a maximum likelihood phylogeny using RAxML v.8.2.11 [88] with a general time reversible nucleotide substitution model [89], four gamma categories for rate heterogeneity, and 100 bootstrap replicates, and visualized using the Interactive Tree of Life program [90].

### Analyzing mutations in core genes

To identify all core SNPs for every pair of genomes, we used the program snp-dists v.0.6.3 (https://github.com/tseemann/snp-dists). We compared mean SNP distances within each of the three largest phylogenetic clusters (SC 3, 6, 9) as well as between strains from the same source (food, human, environment). An ANOVA test implemented in R was performed on each dataset. We also calculated the ratio of the number of nonsynonymous substitutions per non-synonymous site (dN) to the number of synonymous substitutions per synonymous site (dS), which can be used as an indicator of selective pressure acting on a protein-coding gene. To calculate dN/dS (also known as Ka/Ks) of each core gene, we used the kaks function implemented in the R package seqinr [91].

### Inferring the population structure of *C. sakazakii*

Population structure analysis was done as previously described [84]. In summary, we used RhierBAPS [34] to identify distinct genetic clusters of *C. sakazakii* within the broader, more heterogeneous population. The previously generated core genome tree was used as an input in the R package phytools [92] and the SCs were plotted on it using the R packages ggtree [93] and ggplot2 [94]. We used PopPUNK to elucidate the divergence of shared sequence and gene content in a population [24]. PopPUNK compares all possible pairs of genomes by calculating the proportion of shared k-mers of different lengths to determine core and accessory distances, which is used to generate a scatterplot of core and accessory distances which shows the predicted clustering of strains [24].

### Recombination detection

Recombination analysis was done as previously described [84]. In summary, we used (1) PHI test implemented in PhiPack v.1.0 (https://www.maths.otago.ac.nz/~dbryant/software/phimanual.pdf) to determine the statistical likelihood of recombination being present in our dataset [56], (2) SplitsTree v.4.14.8 [57] to identify phylogenetic reticulations, (3) fastGEAR to detect evidence for gene mosaicism in core and shared accessory genes [59], and (4) mcorr to calculate the correlation profile, recombination coverage, mean recombination fragment size and the relative rate of recombination to mutation [58].

### Functional classification of genes

We used PANTHER v.14.1 to analyze functional differences in gene content among sequence groups [42]. PANTHER uses hierarchical annotations from the Gene Ontology (GO) Consortium for functional classifications [95]. We performed comparisons of gene content grouped by SC and by source against a reference list containing all the genes in the pan-genome identified by Roary. We used the GO database v.1.2 and genes were classified according to biological process, molecular function complete, and cellular component. The overrepresentation tests were performed using Fisher’s Exact Test with corrections for false discovery rates.

### Detecting antibiotic resistance and virulence genes

We used ABRicate v.0.8.13 to identify horizontally acquired genes that confer antibiotic resistance and genes that are associated with virulence. ABRicate was used in conjunction with Resfinder database [96] (updated on August 30, 2019) and Virulence Factor Database [97] (updated on August 30, 2019) with default settings. The results were combined into a matrix and plotted against the phylogenetic tree of the genus using R and the R packages ggplot2 [94], ggtree [93], and phytools [92].

## Supplementary information

**Additional file 1: Figure S1.** Core genome mutations in *C. sakazakii*. (a) Pairwise core genome SNP distance within each SC (SCs 3, 6 and 9). (b) Pairwise core genome SNP distance between strains from the same source (food, human, environment). (c) Nonsynonymous and synonymous substitution rates of each core gene.

**Additional file 2: Figure S2.** Genes that are exclusively found in different groups of *C. sakazakii*. (a) Based on SC (b) Based on ecological source (food, human, environmental).

**Additional file 3: Figure S3.** Recombination in the genus *Cronobacter*. (a) Bar plot of the total length of recombined DNA of core and accessory genes per genome calculated using fastGEAR. (b) A phylogenetic network of the core genome generated using SplitsTree. Scale bar represents nucleotide substitutions per site.

**Additional file 4: Table S1.** Accession numbers and genome characteristics of the 285 *Cronobacter* genomes used in this study.

**Additional file 5: Table S2.** ANI values (%) for each pairwise genome comparison of *C. sakazakii* and related species.

**Additional file 6: Table S3.** Number of genes in each gene category of the pan-genome (core, soft core, shell, cloud genes). Shown are values for the entire genus and for each *Cronobacter* species.

**Additional file 7: Table S4.** List of *C. sakazakii* core genes inferred by Roary

**Additional file 8: Table S5.** List of dN, dS and dN/dS for every *C. sakazakii* core gene calculated for every pair of genome. (CSV 244 kb)

**Additional file 9: Table S6.** Accessory genes found exclusively in each sequence cluster

**Additional file 10: Table S7.** Accessory genes found exclusively in each ecological source

**Additional file 11: Table S8.** Functional enrichment of accessory genes calculated by PANTHER

**Additional file 12: Table S9.** Matrix showing the presence or absence of antibiotic resistance and virulence genes identified by ABRicate

**Additional file 13: Table S10.** Recombination parameters calculated by mcorr

**Additional file 14: Table S11.** List of recombined genes in *C. sakazakii* inferred by fastGEAR

**Additional file 15: Table S12.** Total length of recombined DNA in each *Cronobacter* genome

## Data Availability

The datasets analyzed in this study were downloaded from and are available in the GenBank database (https://www.ncbi.nlm.nih.gov/genbank/). Accession numbers are listed in Additional file 4: Table S1.

## Abbreviations

ANI: Average nucleotide identity
NCBI: National center for biotechnology information
PHI: Pairwise homoplasy index
SC: Sequence cluster
SNP: Single nucleotide polymorphisms
SRA: Sequence read archive
ST: Sequence type
T6SS: Type VI secretion system
