# Supplementary figures and images for: Pan-genome diversification and recombination in Cronobacter sakazakii, an opportunistic pathogen in neonates, and insights to its xerotolerant lifestyle
Source: BMC Microbiol. 2019 Dec 27;19:306. doi: 10.1186/s12866-019-1664-7 (PMC6935241; doi:10.1186/s12866-019-1664-7)

**a**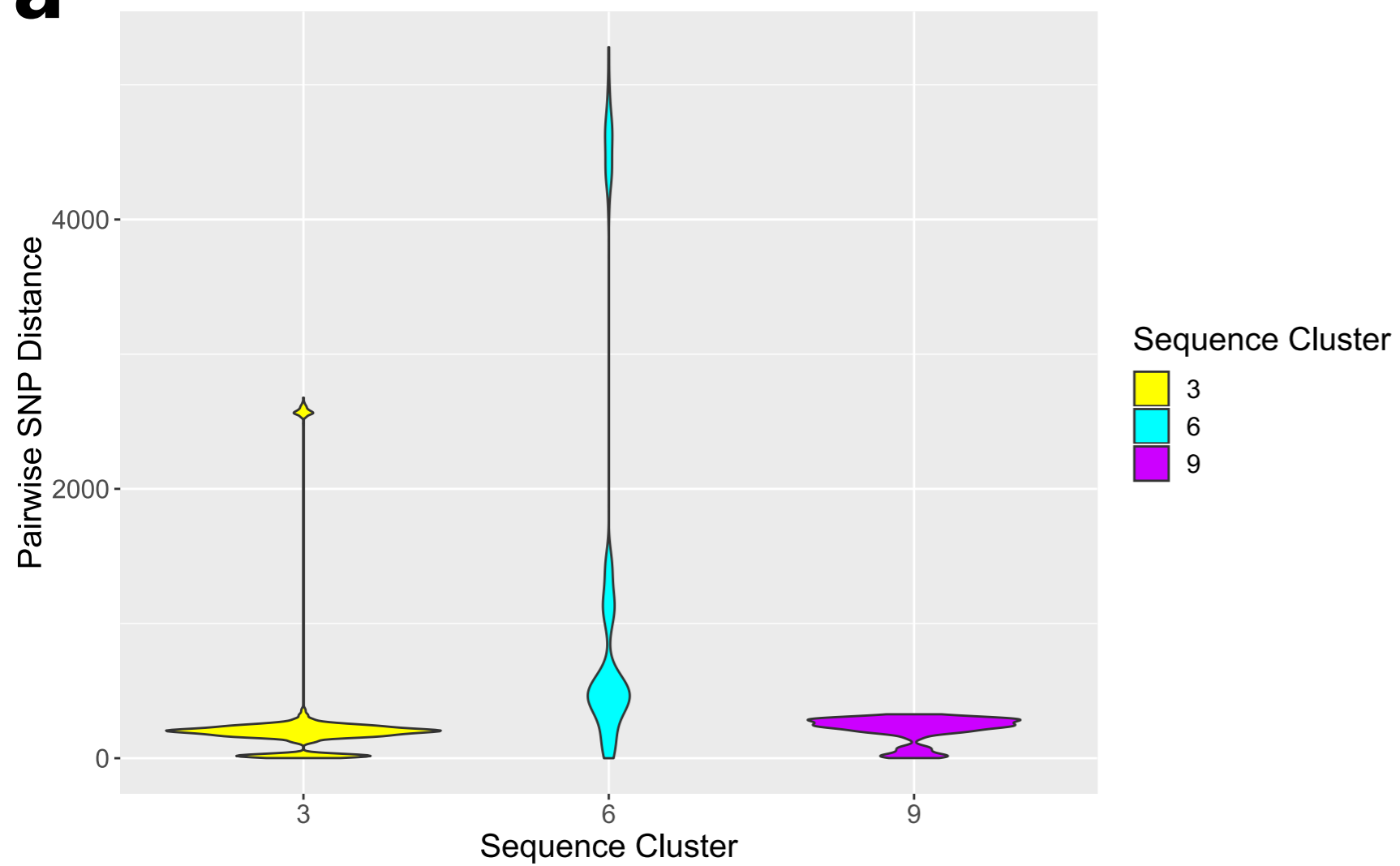**b**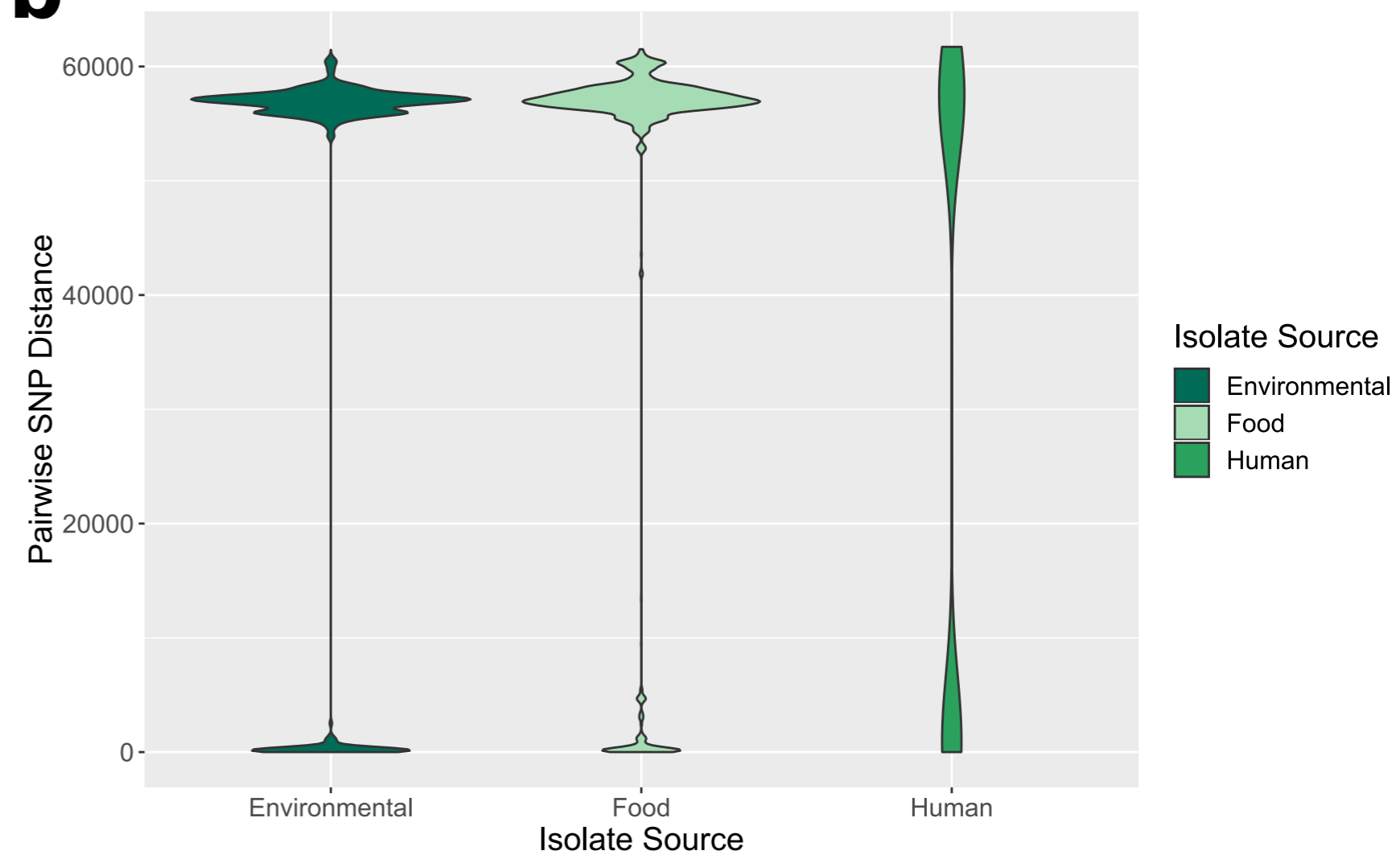**c**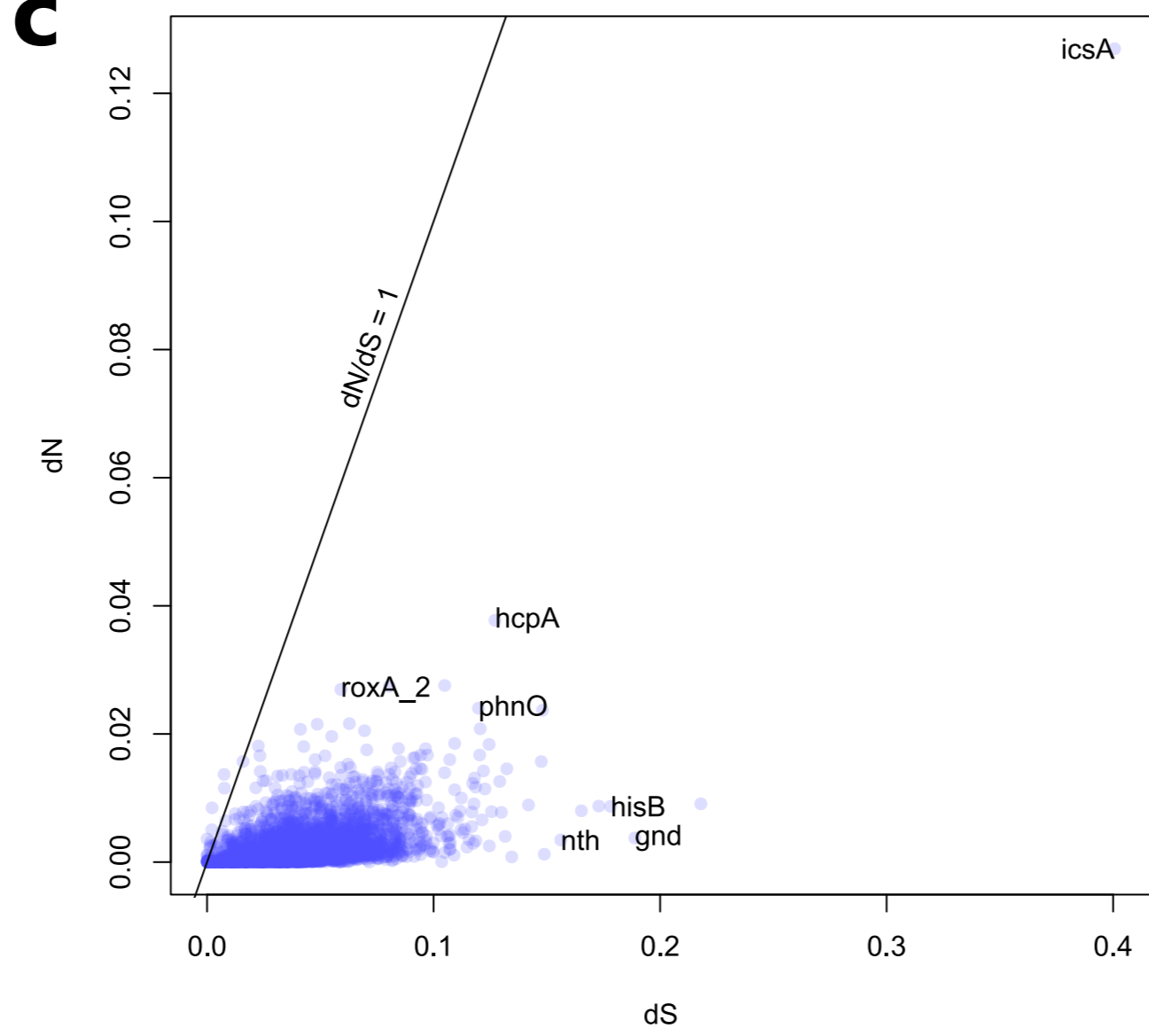

Supplement: Supplementary file 1 — Additional file 1: Figure S1. Core genome mutations in C. sakazakii. (a) Pairwise core genome SNP distance within each SC (SCs 3, 6 and 9). (b) Pairwise core genome SNP distance between strains from the same source (food, human, environment). (c) Nonsynonymous and synonymous substitution rates of each core gene. [file 12866_2019_1664_MOESM1_ESM.pdf]

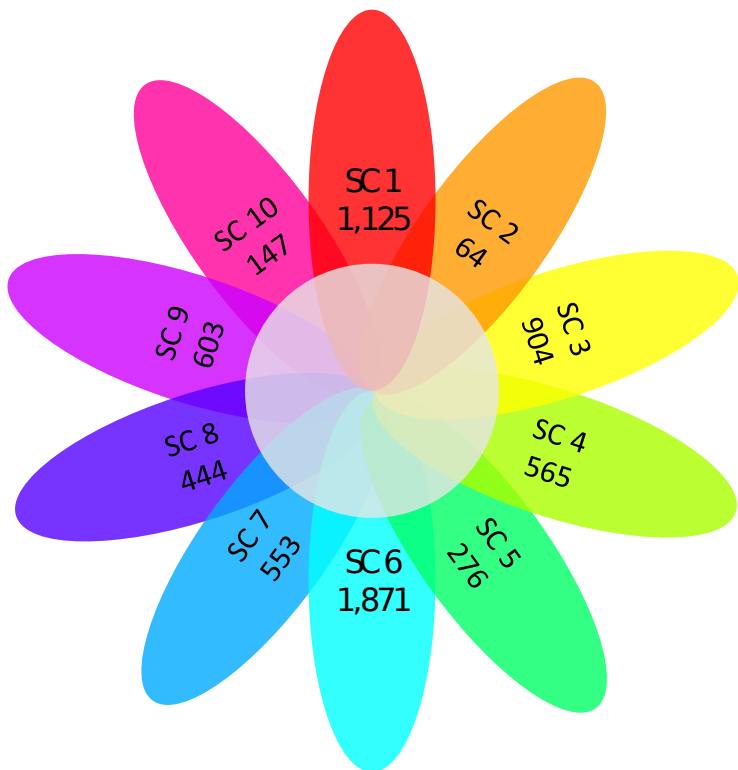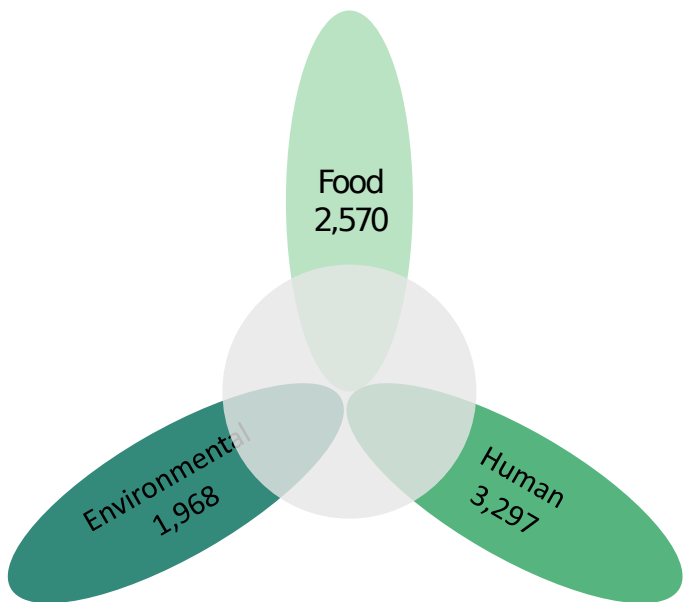

Supplement: Supplementary file 2 — Additional file 2: Figure S2. Genes that are exclusively found in different groups of C. sakazakii. (a) Based on SC (b) Based on ecological source (food, human, environmental). [file 12866_2019_1664_MOESM2_ESM.pdf]

a

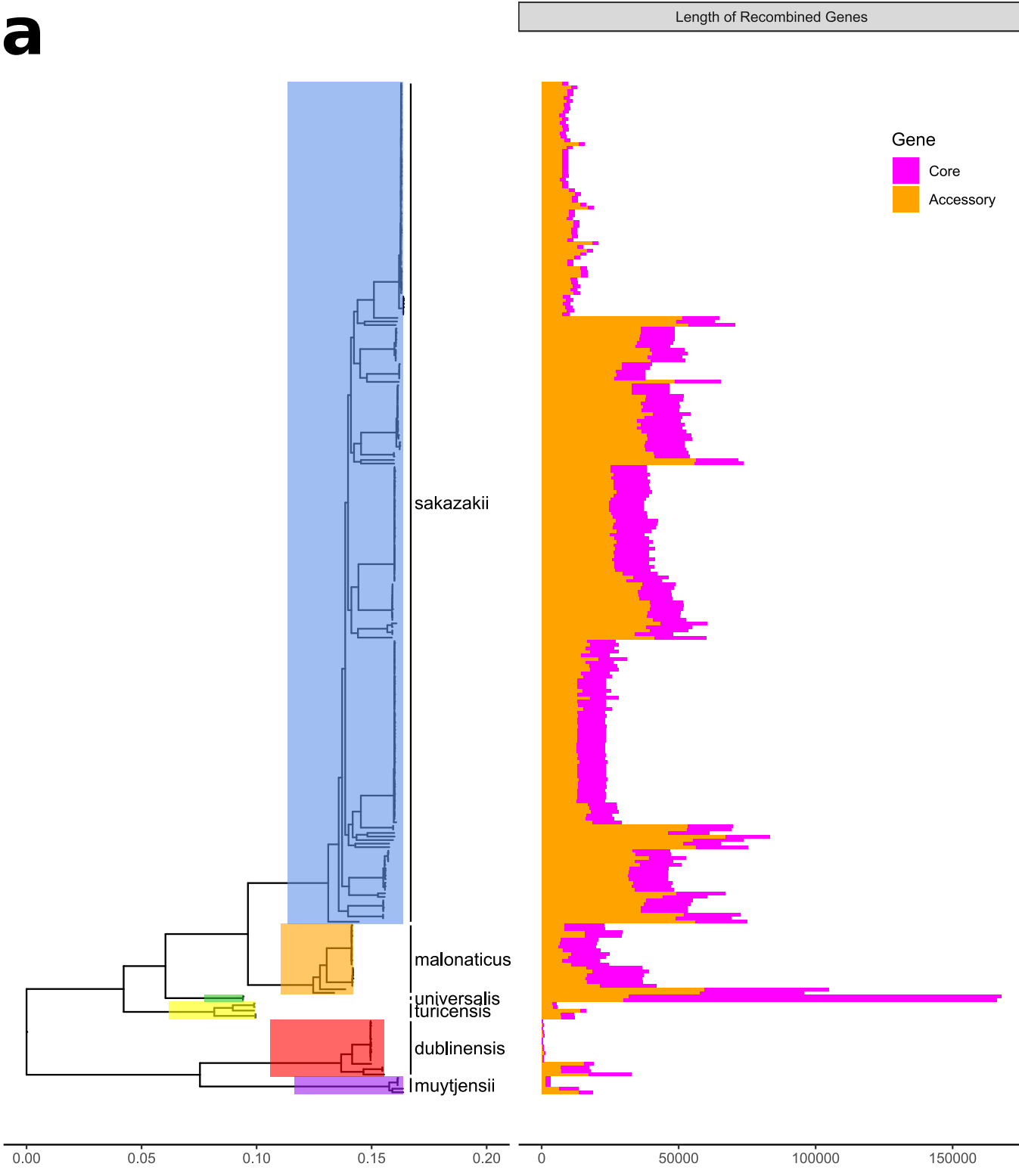

b

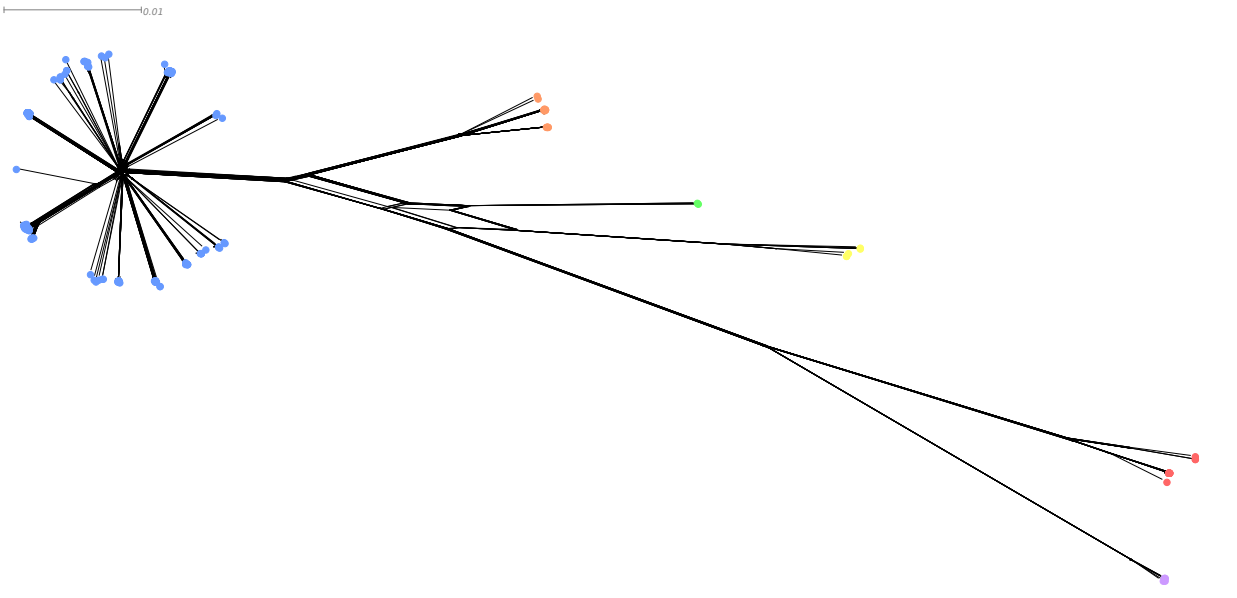

Supplement: Supplementary file 3 — Additional file 3: Figure S3. Recombination in the genus Cronobacter. (a) Bar plot of the total length of recombined DNA of core and accessory genes per genome calculated using fastGEAR. (b) A phylogenetic network of the core genome generated using SplitsTree. Scale bar represents nucleotide substitutions per site. [file 12866_2019_1664_MOESM3_ESM.pdf]
